# Mathematical Model Relating Uniaxial Compressive Behavior of Manufactured Sand Mortar to MIP-Derived Pore Structure Parameters

**DOI:** 10.1155/2014/736230

**Published:** 2014-07-15

**Authors:** Zhenghong Tian, Jingwu Bu

**Affiliations:** College of Water Conservancy and Hydropower Engineering, Hohai University, Nanjing, Jiangsu 210098, China

## Abstract

The uniaxial compression response of manufactured sand mortars proportioned using different water-cement ratio and sand-cement ratio is examined. Pore structure parameters such as porosity, threshold diameter, mean diameter, and total amounts of macropores, as well as shape and size of micropores are quantified by using mercury intrusion porosimetry (MIP) technique. Test results indicate that strains at peak stress and compressive strength decreased with the increasing sand-cement ratio due to insufficient binders to wrap up entire sand. A compression stress-strain model of normal concrete extending to predict the stress-strain relationships of manufactured sand mortar is verified and agreed well with experimental data. Furthermore, the stress-strain model constant is found to be influenced by threshold diameter, mean diameter, shape, and size of micropores. A mathematical model relating stress-strain model constants to the relevant pore structure parameters of manufactured sand mortar is developed.

## 1. Introduction

Natural sand has risen in price and the excessive exploration appears in some area, which will lead to the destruction of the environment and the nature energy crisis. However, manufactured sand produced by rock can meet the strategic requirement of sustainable development and save natural resource to be an alternative to natural sand. Manufactured sand has good characteristics of stable quality, adjustable particle gradation, rough surface, and sharp particle guarantee excellent mechanism of materials prepared by it [1]. In recent years, many researchers [2, 3] initiated studies on cement-based materials prepared by manufactured sand to substitute natural sand. Rough texture, sharp particles and appropriate content of limestone powder in manufactured sand contribute to lower slump and good bleeding and cohesiveness of fresh concrete mixtures [2, 4], which make it easy to vibration and molding. It is demonstrated that the compressive strength of manufactured sand concrete is slightly higher than that of natural sand concrete [3], dramatically in low-strength concrete. For characteristics of durability, limestone powder in manufactured sand can finer pore structure of concrete, which improves permeability as well as freezing and thawing resistance [5, 6]. With excellent mechanism of manufactured sand addition to gradually reduce useable natural sand in China, it will be the best substitute for natural sand as fine aggregate prepared in cement-based materials.

At present, experimental researches on cement-based materials prepared by manufactured sand are restricted to macro mechanical properties [1–6]. The mechanism of manufactured sand on macro mechanical properties (such as bleeding and cohesiveness of fresh mixture, compressive strength and tensile strength, and durability) of cement-based materials is ascribed as the rough texture and sharp particle in the above researches. However, the microstructure and the effect on structural performance of cement-based materials prepared by manufactured sand have received relatively less attention. Cement-based materials contain air voids, capillary pores, and gel pores, and the pores in concrete are randomly sized arranged and connected [7]. The different chemical and physical properties of manufactured sand from these of natural sand are assumed to produce different pore structures in cement-based materials prepared by manufactured sand. Literatures have concentrated on the influence of concrete design parameters [8, 9] such as water-cement ratio, sand-cement ratio, the largest aggregate size, admixture and additives, and test conditions [10–12] on compressive performance. Modeling study was carried out to give a further analytical formulas for mechanical behavior of concrete [13, 14]. While researches [15, 16] concerned less influence of pore structure features on compressive performance of concrete. Generally speaking, strength is inversely related to the porosity for all porous materials [17], so minimized porosity is an available measurement to obtain better material performance. However, it is possible that many of the pore structure features such as total porosity, pore size distribution, and pore shape could be widely different for porous materials [18]. Therefore, it is necessary to account for the contribution of pore structure features on the compressive behaviour of cement-based materials prepared by manufactured sand.

The objective of this research is to develop simplified mathematical models for the interpretation of MIP data and to extract relevant pore structure parameters which can be used for prediction of uniaxial compressive response. Uniaxial compressive response of manufactured sand mortars proportioned using different water-cement ratio and sand-cement ratio is examined so as to bring out the influence of different pore structure features on it. Pore structure features are quantified by using mercury intrusion porosimetry technique. Uniaxial compressive stress-strain relationships are obtained for manufactured sand mortars and are related to pore structure parameters. It is believed that a proper understanding of the influence of pore structure features on compressive stress-strain relationship can lead to optimized material design for the desired properties of manufactured sand mortar.

## 2. Materials and Methods

### 2.1. Materials and Specimen Preparation

Ordinary Portland cement without mineral additions and manufactured sand consisting mainly of calcium carbonate were used as binder and fine aggregate, respectively. The density of cement is 3200 kg/m^3^, and that of manufactured sand is 2650 kg/m^3^. The gradation test showed that the particle size of the manufactured sand was continuously distributed within 0.075–5 mm without limestone powder. In this experimental research, 12 mix proportions of cement mortar with the water-cement ratio of 0.4, 0.5, 0.6, and 0.7 were prepared, and the sand-cement ratios were adjusted according to fluidity of the mixtures, as shown in Table 1. The mixtures were prepared using a laboratory mixer, cast in prism molds, and consolidated using a laboratory vibrator. Then the specimens were kept in the molds for 24 hours. After curing in an environmental chamber at 20°C and 60% relative humidity for 14 days in a laboratory, core drilling machine was used to drill cylinder specimens of 50 mm diameter and 100 mm height, and the specimens ends were ground using a grinding machine and polished in order to ensure smooth and plane surfaces for uniaxial compressive testing. Three electrical resistance strain gauges were pasted on the samples surface along the loading diameter in order to record the strain histories along with stress.

### 2.2. Determination of Compressive Response

**Figure 1:**
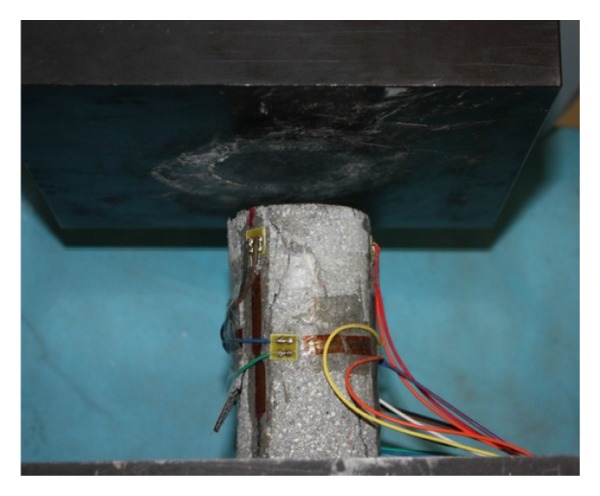
A specimen placed on test setup.

The compressive response of manufactured sand mortars were determined using a 1000 kN closed-loop universal machine operating in uniform loading controlled mode of 1 kN/s according to ASTM C349 [19], and the strain values were collected by using DASP 10.0 data acquisition instrument. Test setup placed a specimen as shown in Figure 1.

### 2.3. Determination of Pore Structure Features

The total porosity of the mortar samples was estimated from the weight of the test sample after drying in an oven at 105°C until reaching constant weight, followed by saturation of the sample by immersion in water for 72 hours, according to the ASTM C642-97 [20]. This allowed the fraction of pore volume to be accessible to water in the mortar. The porosity was calculated using

(1) $$\begin{array}{c} P=\frac{\left(W_{\text{ssd}}-W_{d}\right)}{\left(W_{\text{ssd}}-W_{w}\right)}\times 100\%, \end{array}$$

where *P* is the porosity (%), *W*_ssd_ is mass of the surface-dry sample in air after immersion and boiling (g), *W*_*d*_ is mass of oven-dried sample in air (g), and *W*_*w*_ is apparent mass of sample in water after immersion and boiling (g).

Mercury intrusion porosimetry (MIP) is a widely used method for measuring the pore size distribution of cement-based materials. In MIP test, samples are intruded into a chamber; the chamber is evacuated; the samples are surrounded by mercury and pressure ranging from subambient to 60,000 psi (414 MPa). The contact angle and surface tension of mercury were assumed to be 117° and 0.484 N/m, respectively. On the pressure, the smallest pore size into which mercury can be intruded is 2 nm, and the largest pore size which can be intruded is 200 mm with subambient pressure. The MIP results were obtained in the form of raw data representing cumulative intruded volume versus pore diameter curves and logarithmic differential intruded volume versus pore diameter of cement mortar curves.

## 3. Results and Discussion

### 3.1. Pore Structure Features of Porous Materials

**Figure 2:**
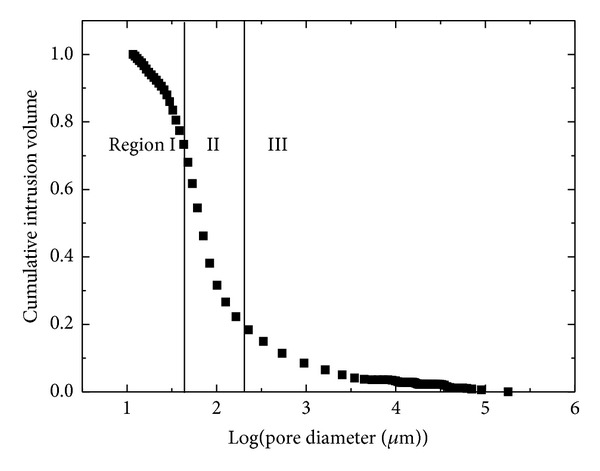
Typical MIP curve divided into three regions.

Pore size distribution as well as some simplified parameters will be developed using MIP technique. Some pore structure parameters from MIP curves for the strength of cement composites seem to be useful from some experimental researches [21]. Pore structure parameters include total porosity [17], pore size distribution [22], and pore shape [16]. According to these parameters, MIP curve can be divided into 3 regions as shown in Figure 2. Regions I, II, and III in MIP curves correspond to macropores, channels interconnecting macropores, and micropores of porous materials, respectively. Therefore, the feasible parameters for macropores, channels, and micropores denoted by *r*_1_, *d*_0_, and *r*_2_, respectively, can be extracted from the corresponding regions. The objective of this paper is to characterize microstructure features and develop a simplified model related compressive response to pore structure parameters. Mathematical model of strength and pore structure features expressed as follows is used in the deducing process:

(2) $$\begin{array}{c} \sigma =f\left(V\right), \end{array}$$

where *f* is the function of pore volume *V*; pore structure features can be characterized by MIP data as follows:

(3) $$\begin{array}{c} V\left(d\right)=\frac{1}{1+\exp\left(\left(d-d_{0}\right)/r_{1}\right)},\;d>d_{0},\\ V\left(d\right)=\frac{1}{1+\exp\left(\left(d_{0}-d\right)/r_{2}\right)},\;d<d_{0}, \end{array}$$

where *V*(*d*) is the cumulative mercury intrusion volume, *d* is logarithm pore diameter, *d*_0_ represents channels interconnecting macropores and is equivalent to logarithm pore diameter at 50% total mercury intrusion volume, and *r*_1_ and *r*_2_ represent curvatures of macropore and micropore region curves, respectively.

Taeun's research [16] on quantified relationships of compressive strength and pore structure features indicated that total porosity *P*, channel pore diameter *d*_0_, and curvatures of macropore region and micropore region, *r*_1_ and *r*_2_, cannot predict compressive strength by statistical analysis precisely; therefore, pore structure parameters should be corrected.

**Figure 3:**
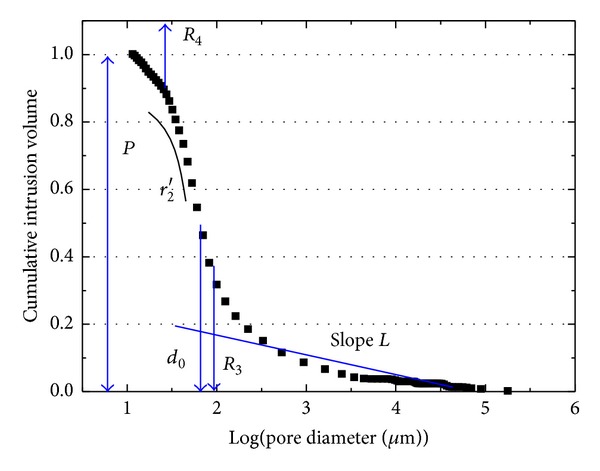
Pore structure parameters shown in a typical MIP cumulative curve.

As mentioned by Carniglia [23], interaction of pores with cracks also must be considered. In this respect, the curvature of the tip of micropores is important parameter. Micropores, therefore, must be characterized with regard to size and shape along with total porosity. For these micropores or cracks, the major concern is their length and eccentricity. Both *R*_4_ equal to 90% total intrusion volume of MIP cumulative curve and *r*_2_′ representing pores shape instead of *r*_2_ will be studied in the following parts. It is difficult to characterize macropore shape using MIP data; however, it is easy to obtain the slope *S*_*L*_ of macropore region as the amount of macropores substitute curvature of macropore region *r*_1_. As for channels, *d*_0_ and *R*_3_ are selected as alternatives to *d*_0_, which cannot reflect major characteristics of channels in reality. Threshold diameter *R*_3_ can be defined as the pore diameter at which the largest mercury intrusion during MIP occurs; at this point the negative curvature of the cumulative mercury intrusion volume curve turns to be positive; usually this is not the same as *d*_0_. *R*_3_ is another new microstructure parameter for characterizing the major threshold. For the above background of micropore, macropore, and threshold, a new set of pore structure parameters, *P*, *S*_*L*_, *d*_0_, *R*_3_, *R*_4_, and *r*_2_′ are formulated. Graphically these parameters are shown in Figure 3.

### 3.2. Pore Structure Characteristics of Manufactured Sand Mortar

**Figure 4:**
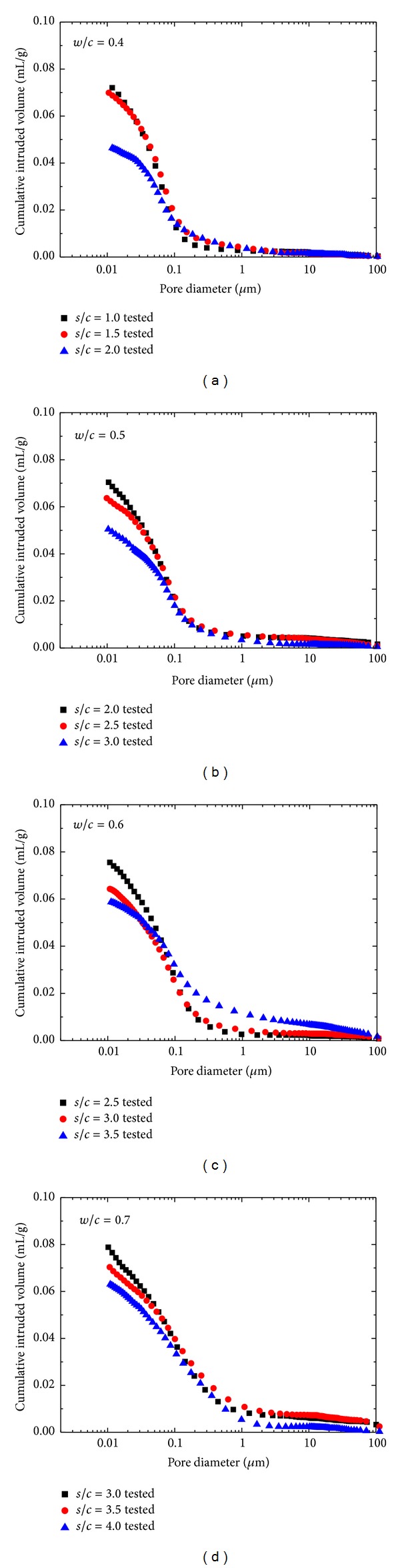
Cumulative intrusion volume versus pore diameter curves for all mix proportions of manufactured sand mortars.

In this section, the pore size distributions of manufactured sand mortars with different mix proportions after curing 28 days were discussed. The cumulative intrusion volume versus logarithm pore diameter curves is shown in Figure 4.

The MIP data indicated a threshold diameter below which there is a relatively little intrusion and immediately above which rapid intrusion commences. This corresponds to the threshold region, namely, Region II of inflection, following an almost horizontal portion of cumulative intrusion curves as shown in Figure 2. Cumulative curves of manufactured sand mortars in Figure 4 showed that the threshold diameter increases with increasing sand-cement ratio and water-cement ratio. If threshold diameter is assumed to be the initial intergranular spacing at the setting time, the higher water-cement ratio generates a higher threshold diameter. As sand-cement ratio increases, the major threshold region becomes flattened out and threshold diameter increases (65 nm–97 nm) progressively, which can be attributed to fine aggregate effect of reorientation of pore system of mortar. Different from cement paste, the threshold diameter in mortar is linked to the binder-aggregate interface or even fissures rather than to the pores alone [24]. Okpala [25] demonstrated that the total intruded pore volume decreased with increasing aggregate volume concentration. However intruded pore volume per volume of paste in the mortar increases with increase in aggregate volume concentration. This strongly suggested that pores being intruded by mercury may not be pores in the paste alone but could include fissures and bond cracks at the aggregate-paste interface.

Pore structure parameters for each composition are summarized in Table 2 and used in the multiple linear regression for compressive response of manufactured sand mortar. The fundamental objective of this paper is to relate uniaxial compressive response of manufactured sand mortar to its pore structure parameters. This will be realized by determination of stress-strain relationship of several manufactured sand mortars and modeling the stress-strain relationship as a function of pore structure parameters through statistical analysis. These following sections solve these aspects in detail.

### 3.3. Stress-Strain Relationships for MS Mortars

**Figure 5:**
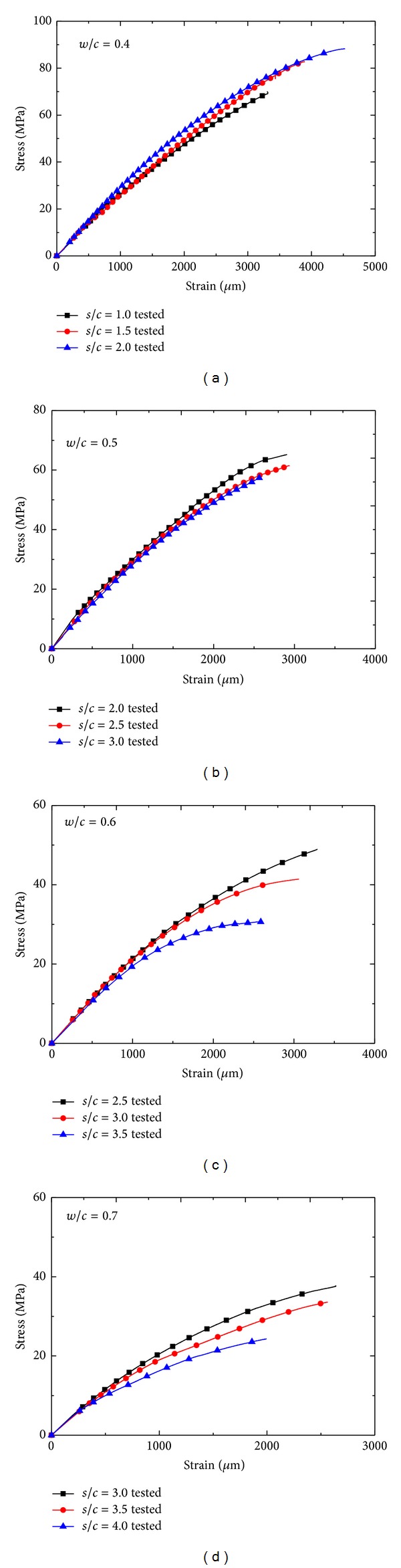
Typical uniaxial compressive stress-strain curves for all different mix proportions of manufactured sand mortars.

The typical uniaxial compressive stress-strain curves for all mix proportions of manufactured sand mortars are shown in Figures 5(a)–5(d). These stress-strain curves of three specimens belong to mixtures with same *w*/*c*. Strains at peak stresses coming along compressive strength decrease with sand-to-cement ratio increasing in all specimens except specimens with 0.4 *w*/*c*. The decreasing compressive strength of specimens with 0.5, 0.6, and 0.7 *w*/*c* is lower than that of specimens with 0.4 *w*/*c*; the relatively lower compressive strength values can be attributed to higher *w*/*c* and sand-cement ratio in these mixtures, in which there is no sufficient inclusion binder of manufactured sand. The influence of *w*/*c* and sand concentration on porosity is that porosity increases with increasing *w*/*c* and decreasing sand content. As mentioned above, porosity cannot predict compressive strength with confidence. Microstructural parameters including total porosity, pore size, and pore shape should be taken into consideration to develop mathematical relationships between compressive response and pore structure parameters.

### 3.4. Model for Stress-Strain Relationships of MS Mortars

**Figure 6:**
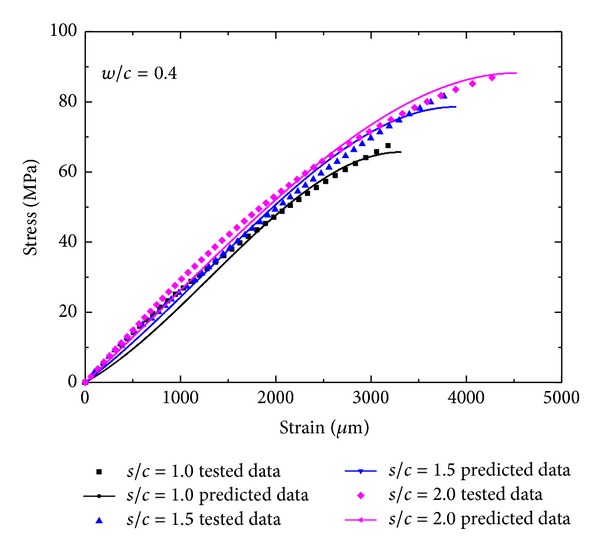
Typical stress-strain relationship fitting results of manufactured sand mortar.

In the past, many studies [26, 27] have proposed stress-strain relationships for the normal concrete prepared by a wide variety of mixture design parameters and material inclusions. Stress-strain relationships are usually influenced by material parameters and testing conditions. For concrete materials, aggregate-paste interface is considered to be the weakest region. Stress-strain curves are influenced mainly by testing conditions, such as loading rate and specimen shape and size, and material parameters including water-cement ratio and the largest aggregate size. It is relatively difficult to obtain the stress-strain curves of concrete in comparison to compressive strength and elastic modulus, which is easy to be acquired. Some researchers tried to examine compressive strength and elastic modulus to predict the stress-strain relationships. The objective of this study is to develop the simplified model of compressive response and pore structure features. The foregoing sections have determined pore structure features by using MIP data, and the following section will build stress-strain model of manufactured sand mortar according to the conventional concrete stress-strain models and establish the model parameters meanwhile. In this paper, the analytical model proposed by Guo and Zhang [28] for uniaxial compression of normal concrete is used. This expression model was developed for normal concrete and is extended in this research on manufactured sand mortar. The normalized stress-strain relationship of manufactured sand mortar is expressed as follows:

(4) $$\begin{array}{c} \overset{-}{\sigma }=a\overset{-}{\varepsilon }+\left(3-2a\right){\overset{-}{\varepsilon }}^{2}+\left(a-2\right){\overset{-}{\varepsilon }}^{3},\;\text{for}\;\;\overset{-}{\varepsilon }<1, \end{array}$$

where $\overset{-}{\varepsilon }=\varepsilon /\varepsilon_{0}$, $\overset{-}{\sigma }=\sigma /f_{c}$, *f*_*c*_ is compressive strength, *ε*_0_ is the strain at peak stress, and *a* is fitting constant. The parameter *a* reflecting the shape of the ascending portion of the stress-strain relation can be simplified to be approximately linear, and so the relationship between *a* and pore structure features can be also considered to be linear.  Figure 6 shows the typical stress-strain fitting results of manufactured sand mortar.

### 3.5. Relationships between Stress-Strain Curves and Pore Structure Features

In this section, analytical model characterized stress-strain curve model parameter *a* and pore structure features. Table 2 lists the values of pore structure parameters acquired by using MIP data of all mix proportions of manufactured sand mortar. The relationship between *a* and pore structure features can be considered to be linear as mentioned previously. The multiple linear regression model is expressed as follows:

(5) $$\begin{array}{c} a=\underset{i}{\sum }B_{i}X_{i}, \end{array}$$

where *X*_*i*_ represent pore structure features, *P*, *S*_*L*_, *d*_0_, *R*_3_, *R*_4_, *r*_2_′. *B*_*i*_ is the fitting coefficient. Statistics of these pore structure parameters and stress-strain curve model parameter *a* is summarized in Table 3. The correlation matrix in Table 3 reveals the general correlation between the dependent variable, *a*, and independent variables, pore structure parameters. Regarding the correlation between independent and dependent variables, the common feature is that all parameters are positively correlated to the stress-strain model parameter *a*. Among these parameters, *r*_2_′, *R*_3_, and *d*_0_ have high correlation coefficients greater than 0.7; this high correlation means that these three parameters should be entered in the statistical analysis. Although the correlation matrix indicated the significance of *r*_2_′, *R*_3_, and *d*_0_, the selection of microstructural parameters as the independent variables must be completed with selection criteria such as *R* ^2^ and AIC (Aikake's information criterion). There are 10 cases for selection of pore structure parameters listed in Table 4. The results reveal that the more independent variables are, the higher the values of *R* ^2^, which demonstrates that the increase in the number of independent variables can enhance the correlation between dependent variables and independent variables. Compared to case 2, case 5 has the additional independent parameter *P*, which does not increase *R* ^2^ that indicates *P* is not significant to dependent variable *a*. However, in case 7, *R*_3_ and *R*_4_ increase *R* ^2^, which means that these parameters are highly significant for the dependent variable *a*. Therefore, the best model is case 10 according to the criteria of the highest *R* ^2^ and the least AIC. The result of regression analysis with parameters, *d*_0_, *R*_3_, *R*_4_, *r*_2_′, are shown in Table 5. Researches on the relations of compressive strength and microstructural parameters indicated that total porosity is significant to compressive behavior [11]; nevertheless, it is insignificant for uniaxial compressive response of manufactured sand mortar by statistical analysis in this paper. The parameter for amount of macropores *S*_*L*_ is necessary to the independent variable *a*. Pore structure parameters, *d*_0_ and *R*_3_, which reflect the characteristics of channels, as well as *R*_4_ and *r*_2_′, which represent the size and shape of micropore, respectively, are important factors for compressive response.

A multiple nonlinear regression model was found to provide a higher degree of predictive accuracy by Deo and Neithalath [15], and when all pore structure parameters were considered in the multiple linear regression model, issue of multicollinearity was observed. Therefore, measurement must be taken to avoid multicollinearity by modifying model terms; this study was based on grouping the individual pore structure parameters so as to reduce the intercorrelation between independent variables. *d*_0_ and *R*_4_ are equivalent to 50% and 90% of total mercury intrusion volume, respectively, so *d*_0_/*R*_4_ can be used to characterize the size of micropores to channels. *R*_3_ represents the major threshold as a single term. *r*_2_′, which can reflect the shape and size of micropores, is the most significant parameter to dependent variable, and there is no multicolinearity phenomenon between this and any other independent variables as shown in Table 3. The model and model terms were further modified by appropriate transformations so as to avoid multicolinearity phenomenon and obtain the best possible improvements in the model *R* ^2^. The final model terms were determined as ln⁡(*r*_2_′), ln⁡(*R*_4_)/ln⁡(*d*_0_), and *R*_3_. Correlation matrix of modified model terms for compressive response is listed in Table 5. And Table 6 summarizes all models results; the modified model *R* ^2^ was improved, and the issue of muticolinerity was solved. The modified model is expressed as follows:

(6) $$\begin{array}{c} a=B_{0}+B_{1}\ln\left(r_{2}^{\prime }\right)+B_{2}\left(\frac{\ln\left(R_{4}\right)}{\ln\left(d_{0}\right)}\right)+B_{3}R_{3}. \end{array}$$

**Figure 7:**
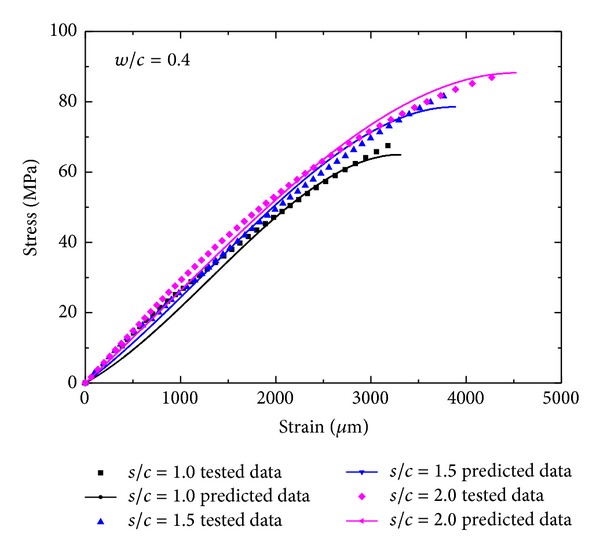
Experimental data and model fits for the stress-strain relationship by pore structure parameters of MS mortar.

Backwards simulation for stress-strain curves using the calculated seems to be fitted well with tested data as shown in Figure 7, which demonstrates the multiple nonlinear regression model has higher prediction accuracy.

## 4. Conclusions

The primary objective of this paper is to develop a simplified mathematical model relating compressive stress-strain relationship to its relevant pore structure parameters other than total porosity derived by MIP technique. Experiments and statistical analysis were carried out and the following fundamental conclusions can be drawn:

- threshold diameter increases with increasing sand-cement ratio and water-cement ratio. The threshold region (Region II) becomes flattened and horizontal along with increasing sand concentration in manufactured sand mortar; this is mainly because of the reorientation effect of fine aggregates on pore structure;
- strains at peak stresses and compressive strength of manufactured sand mortar decrease with increasing water-cement ratio and sand-cement ratio in all specimens except for that, with 0.4 w/c, the relatively lower compressive strength values can be attributed to higher w/c and sand-cement ratio in these mixtures, in which there is no sufficient inclusion binder of manufactured sand;
- a model proposed for the stress-strain relationship of normal concrete was found in accordance with experimental data of manufactured sand mortar with a higher precision. The model constant was related to pore structure parameters, while threshold diameter, mean diameter, size, and shape of micropores are responsible for it other than porosity and total amounts of macropores. A mathematical model was developed relating compressive response to relevant pore structure parameters of manufactured sand mortar.

## Figures and Tables

**Table 1:** The mix proportion of 12 manufactured sand mortars.

Specimens	*w*/*c*	*s*/*c*	Fluidity (mm)
M1	0.4	1.0	257.5
M2	0.4	1.5	215.0
M3	0.4	2.0	162.5
M4	0.5	2.0	257.5
M5	0.5	2.5	187.5
M6	0.5	3.0	150.0
M7	0.6	2.5	265.0
M8	0.6	3.0	237.5
M9	0.6	3.5	150.0
M10	0.7	3.0	267.5
M11	0.7	3.5	242.5
M12	0.7	4.0	177.5

**Table 2:** Pore structure parameters for all mix proportions of manufactured sand mortars.

Specimens	*P* (%)	*S*_*L*_	*R*_3_ (*μ*m)	*d*_0_ (*μ*m)	*R*_4_ (*μ*m)	*r*_2_′
M1	0.2213	0.018	0.065	0.055	0.017	0.464
M2	0.2003	0.027	0.067	0.062	0.019	0.382
M3	0.1845	0.042	0.065	0.066	0.025	0.311
M4	0.2213	0.023	0.076	0.062	0.017	0.419
M5	0.2119	0.036	0.078	0.071	0.02	0.357
M6	0.1892	0.03	0.079	0.075	0.018	0.388
M7	0.2241	0.014	0.085	0.071	0.018	0.378
M8	0.2049	0.027	0.088	0.075	0.02	0.344
M9	0.2116	0.084	0.093	0.109	0.027	0.252
M10	0.2365	0.039	0.095	0.095	0.016	0.37
M11	0.221	0.062	0.096	0.121	0.018	0.316
M12	0.2145	0.026	0.097	0.116	0.021	0.296

**Table 3:** Intercorrelation matrix of model terms for compressive response.

Model terms	*a*	*P* (%)	*S*_*L*_	*R*_3_ (*μ*m)	*d*_0_ (*μ*m)	*R*_4_ (*μ*m)	*r*_2_′
*a*	1	0.19	0.63	0.75	0.70	0.56	−0.81
*P* (%)		1	−0.04	0.48	0.28	−0.51	−0.21
*S*_*L*_			1	0.42	0.64	0.62	−0.74
*R*_3_ (*μ*m)				1	0.88	0.02	−0.57
*d*_0_ (*μ*m)					1	0.25	−0.74
*R*_4_ (*μ*m)						1	0.78
*r*_2_′							1

**Table 4:** List of model selection criteria, *R* ^2^, AIC coefficient for combination of pore structure parameters.

Case	*r*_2_′	*R*_3_	*d*_0_	*S*_*L*_	*R*_4_	*P*	*R* ^2^	AIC
1	∗						0.66	−7.6
2	∗	∗					0.78	−11.1
3	∗		∗				0.68	−6.5
4	∗	∗	∗				0.84	−12.7
5	∗	∗		∗			0.79	−9.2
6	∗	∗			∗		0.87	−15.1
7	∗	∗				∗	0.81	−10.6
8	∗	∗	∗			∗	0.88	−13.9
9	∗	∗	∗	∗			0.87	−12.8
10	∗	∗	∗		∗		0.89	−15.6

**Table 5:** Intercorrelation matrix of modified model terms for compressive response.

Model terms	*a*	ln⁡(*r*_2_′)	ln⁡(*R*_4_)/ln⁡(*d*_0_)	*R*_3_ (*μ*m)
*a*	1	−0.82	0.52	0.75
ln⁡(*r*_2_′)		1	−0.45	−0.56
ln⁡(*R*_4_)/ln⁡(*d*_0_)			1	0.89
*R*_3_ (*μ*m)				1

**Table 6:** Coefficients for multiple linear model and multiple nonlinear model.

Model terms	*B*_0_	*B*_1_(*P*)	*B*_2_(*S*_*L*_)	*B*_3_(*R*_3_)	*B*_4_(*d*_0_)	*B*_5_(*R*_4_)	*B*_6_(*r*_2_′)	*R* ^2^
Coefficient	−1.7	5.1	1.9	27.2	−9.0	54.3	−1.6	0.92

Model terms	*B*_0_	*B*_1_(*r*_2_′)	*B*_2_(*R*_3_)	*B*_3_(*d*_0_)	*B*_4_(*R*_4_)	*R* ^2^	AIC	

Coefficient	−2.01	−0.04	32.11	−6.80	67.03	0.89	−15.62	

Model terms	*B*_0_	*B*_1_(ln⁡(*r*_2_′))	*B*_2_(ln⁡(*R*_4_)/ln⁡(*d*_0_))	*B*_3_(*R*_3_)	*R* ^2^	AIC		

Coefficient	−0.17	−1.14	−1.29	28.96	0.91	−16.10		

## References

[1] Wakchaure MR, Shaikh AP, Gite BE (2012). Effect of types of fine aggregate on mechanical properties of cement concrete. *International Journal of Modern Engineering Research*.

[2] Cortes DD, Kim H-K, Palomino AM, Santamarina JC (2008). Rheological and mechanical properties of mortars prepared with natural and manufactured sands. *Cement and Concrete Research*.

[3] Gonçalves JP, Tavares LM, Toledo Filho RD, Fairbairn EMR, Cunha ER (2007). Comparison of natural and manufactured fine aggregates in cement mortars. *Cement and Concrete Research*.

[4] Nanthagopalan P, Santhanam M (2011). Fresh and hardened properties of self-compacting concrete produced with manufactured sand. *Cement and Concrete Composites*.

[5] Li B, Ke G, Zhou M (2011). Influence of manufactured sand characteristics on strength and abrasion resistance of pavement cement concrete. *Construction and Building Materials*.

[6] Bhikshma V, Kishore R, Patchi CVR (2010). Investigations on flexural behavior of high strength manufactured sand concrete. *Challenges, Opportunities and Solutions in Structural Engineering and Construction*.

[7] Diamond S (1971). A critical comparison of mercury porosimetry and capillary condensation pore size distributions of portland cement pastes. *Cement and Concrete Research*.

[8] Ahmad SH, Shah SP (1982). Stress-strain curves of concrete confined by spiral reinforcement. *ACI Journal Proceedings*.

[9] Wee TH, Chin MS, Mansur MA (1996). Stress-strain relationship of high-strength concrete in compression. *Journal of Materials in Civil Engineering*.

[10] Chen X, Wu S, Zhou J (2014). Strength values of cementitious materials in bending and in tension. *ASCE Journal of Materials in Civil Engineering*.

[11] Chen X, Wu S, Zhou J (2014). Quantification of dynamic tensile behavior of cement-based materials. *Construction and Building Materials*.

[12] Chen X, Wu S, Zhou J, Chen Y, Qin A (2013). Effect of testing method and strain rate on stress-strain behavior of concrete. *Journal of Materials in Civil Engineering*.

[13] Chen X, Wu S, Zhou J (2013). Experimental and modeling study of dynamic mechanical properties of cement paste, mortar and concrete. *Construction and Building Materials*.

[14] Chen X, Wu S, Zhou J (2013). Experimental study and analytical formulation of mechanical behavior of concrete. *Construction and Building Materials*.

[15] Deo O, Neithalath N (2010). Compressive behavior of pervious concretes and a quantification of the influence of random pore structure features. *Materials Science and Engineering A*.

[16] Taeun P (1993). *Development of analytical model relating compressive strength in porous ceramics to MIP-derived microstructural parameters [Ph.D. thesis]*.

[17] Chen X, Wu S, Zhou J (2013). Influence of porosity on compressive and tensile strength of cement mortar. *Construction and Building Materials*.

[18] Neithalath N, Sumanasooriya MS, Deo O (2010). Characterizing pore volume, sizes, and connectivity in pervious concretes for permeability prediction. *Materials Characterization*.

[19] ASTM C349 Standard test method for compressive strength of hydraulic cement mortars.

[20] ASTM C642 (2002). *Standard Test Method for Density, Absorption and Voids in Hardened Concrete*.

[21] Jambor J (1990). Pore structure and strength development of cement composites. *Cement and Concrete Research*.

[22] Chen X, Wu S (2013). Influence of water-to-cement ratio and curing period on pore structure of cement mortar. *Construction and Building Materials*.

[23] Carniglia SC (1972). Working model for porosity effects on the uniaxial strength of ceramics. *Journal of the American Ceramic Society*.

[24] Feldman RF (1986). The effect of sand/cement ratio and silica fume on the microstructure of mortars. *Cement and Concrete Research*.

[25] Okpala DC (1989). Pore structure of hardened cement paste and mortar. *International Journal of Cement Composites and Lightweight Concrete*.

[26] Almusallam TH, Alsayed SH (1995). Stress-strain relationship of normal, high-strength and lightweight concrete. *Magazine of Concrete Research*.

[27] Hsu LS, Hsu CTT (1994). Complete stress—strain behaviour of high-strength concrete under compression. *Magazine of Concrete Research*.

[28] Guo ZH, Zhang XQ (1982). Experimental investigation of stress-strain curves for concrete, Chinese. *Journal of Building and Structure*.

